# Identifying developments over a decade in the digital health and telemedicine landscape in the UK using quantitative text mining

**DOI:** 10.3389/fdgth.2023.1092008

**Published:** 2023-04-17

**Authors:** Nophar Geifman, Jo Armes, Anthony D. Whetton

**Affiliations:** ^1^ School of Health Sciences, Faculty of Health and Medical Sciences, University of Surrey, Guildford, United Kingdom; ^2^ School of Biosciences and Medicine, Faculty of Health and Medical Sciences, University of Surrey, Guildford, United Kingdom; ^3^ Veterinary Health Innovation Engine, School of Veterinary Medicine, University of Surrey, Guildford, United Kingdom

**Keywords:** digital health, telemedicine, United Kingdom, trends, text mining

## Abstract

The use of technologies that provide objective, digital data to clinicians, carers, and service users to improve care and outcomes comes under the unifying term Digital Health. This field, which includes the use of high-tech health devices, telemedicine and health analytics has, in recent years, seen significant growth in the United Kingdom and worldwide. It is clearly acknowledged by multiple stakeholders that digital health innovations are necessary for the future of improved and more economic healthcare service delivery. Here we consider digital health-related research and applications by using an informatics tool to objectively survey the field. We have used a quantitative text-mining technique, applied to published works in the field of digital health, to capture and analyse key approaches taken and the diseases areas where these have been applied. Key areas of research and application are shown to be cardiovascular, stroke, and hypertension; although the range seen is wide. We consider advances in digital health and telemedicine in light of the COVID-19 pandemic.

## 1. Introduction

Digital health is inclusive of mobile health, health information technology, wearable devices, telehealth and telemedicine, as well as personalised medicine, as defined by the US Food and Drug Administration (1). Each of these areas has an upward trajectory in terms of research interest, commercialisation, and potential application in improving healthcare (2). This is recognised in the release of an FDA Digital Health Innovation Action plan (https://www.fda.gov/media/106331). The realisation that digital solutions can deliver support and real impact for healthcare, patient management and outcomes, as well as medical research has been a driver for new adoption of technologies (3, 4).

However, the field is broad, rapidly changing and it is not immediately obvious what directions have been fully explored and what successes have been achieved (5, 6). Whilst the scientific and medical literature offers a clear indication of the development and evidence-based usage of digital health, reviewing the scope of the evidence is traditionally labour-intensive and time consuming. The literature is often fragmented to studies on specific diseases or conditions. Text mining, offers a swift and systematic method to review and better understand the scope of how digital health will alter the health economy and medical outcomes. By taking an objective, data-driven approach to reviewing the vast amount of literature on the subject, a better understanding of the underlying trends can be obtained. Here, using text mining we analyse data derived from publications to gain insight into areas where the United Kingdom (UK) is involved and prolific within digital health.

## 2. Aim

The aim of this review was to identify the range of the published evidence on the use of digital health technology in the UK. The nature of this multidisciplinary field, publishing in many different learned journals, requires the use of an objective tool for data harvesting in order to see trends and growth areas.

## 3. Methods

We have previously described an approach (7, 8) that can mine PubMed records for associations and linkages between different, formally defined, concepts and fields using Medical Subject Heading (MeSH) terms. This quantitative approach is a rapid tool that can be used iteratively to search for trends. PubMed has the broadest range of healthcare and biomedical journal coverage and as such is sufficient as the sole database for use here. Here we apply this approach using digital health related terminology as well as specific diseases. Using an automated script (7), for each PubMed record the list of associated MeSH descriptors was captured. Lists of diseases [extracted from (9)], digital health related terminology (manually compiled by a domain expert and based on literature searches, see below), gender, as well as MeSH-defined age groups, were then searched for exact matches within the MeSH descriptors associated with each PubMed record. Next, co-occurrences of terms were searched for in each PubMed record and counted. These associations and co-occurrences were then used for the analyses described below.

To capture the type of digital approaches, methods and applications used in each search PubMed record, the following MeSh terms were captured: Remote sensing technology, remote consultation, remote monitoring, telerehabilitation, digital divide, mobile applications, telemedicine, internet, smartphone, algorithms, electronic health records, medical informatics, software, user-computer interface, machine learning, artificial intelligence, text messaging, telephone, cell phone, self-management, medical informatics applications, wearable electronic devices, information dissemination, videoconferencing, digital technology, and population surveillance. The criterion for selecting these terms is common usage in the digital health industry, [see for example (10, 11)].

### 3.1. PubMed data

**Figure 1:**
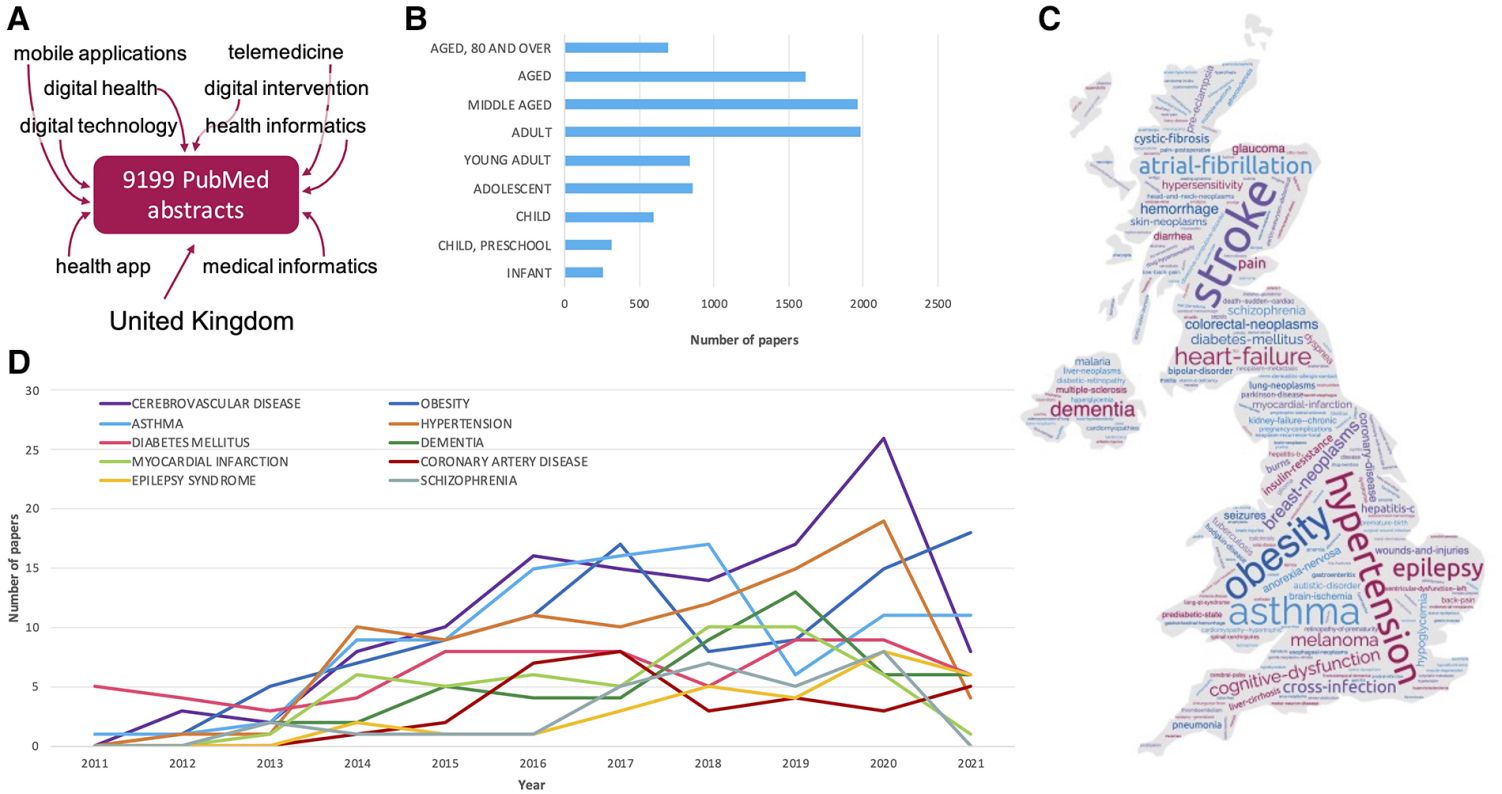
Mining PubMed records for disease-related association with digital-health related publications. (**A**) Terms used to search PubMed for digital health related publications. (**B**) Number of papers associated with different age groups. Age group definitions are derived from MeSH as follows: Infants 1 to 23 months; Child, preschool: 2–5 years; Child: 6–12 years; Adolescent: 13–18 years; Adult: 19–44 years; Middle aged: 45–64 years; Aged: 65–79 years; Aged, 80 and over: 80 years or over. (**C**) Word cloud of the disease terms found in PubMed records associated with digital health; the size of the word corresponds to the number of times it was mentioned across abstracts. The locations of the words on the map do not represent a link to specific UK regions. (**D**) Longitudinal counts of the most prevalent diseases (within the analysed corpus), over the last 11 years.

The inception of the study was a PubMed database search on December 27th, 2021 for publications related to digital health. The search strategy was to identify publications tagged with any of the following terms: “Mobile applications”, “digital health”, “telemedicine”, “digital intervention”, “health app”, “medical informatics”, “health informatics”, or “digital technology”; publications had to have been published between January 1st 2011 up until the date of searching (December 27th 2021)—capturing a timespan of just over a decade. These dates were chosen to highlight current and developing trends. Resulting publications were restricted to those with at least one United Kingdom (or UK) affiliation, as well as having an available abstract. While the approach does not guarantee that a UK organisation/institution has led the research captured within the publication, it captures all those publications that are at least aligned to the UK. The exact search used can be found in Supplementary Material. The search resulted in a total of 9,199 records (Figure 1A).

### 3.2. Network analysis

Network analysis was carried out using the Cytoscape software (12). Cytoscape was used as it is an open source, free tool for modelling and visualising complex interaction networks (13, 14). A network was created using the information extracted from PubMed (as described above); where each MeSH term (disease, or digital health related term) is represented as a node, and an edge between two nodes represents co-occurrence of the two terms within the same PubMed abstracts. Node and edge attributes such as, node type, node counts (of occurrences) and edge counts (of co-occurrences) were used for colouring, sizing, and the layout of the networks. Sub-networks were created by selecting nodes of interest and then selecting first-degree neighbours. The data used to generate the network, as well as a high-resolution full network image are available in Supplementary Material.

## 4. Results

### 4.1. Disease areas of focus for digital health solutions

Within our analysed corpus of published work, cerebrovascular disease was the most prevalent medical condition reported in digital-health related publications (*n* = 119, Figure 1). This was followed by (in descending order) obesity (*n* = 100), asthma (*n* = 98), hypertension (*n* = 92), diabetes (*n* = 69), dementia (*n* = 51), myocardial infarctions (*n* = 50), coronary artery disease (*n* = 33), epilepsy syndrome (*n* = 30), and schizophrenia (*n* = 30). Reports on digital health research and applications related to cerebrovascular disease peaked in 2020, with a reduction in the number of publications in the following year (Figure 1D), though this may be a temporary dip in the trend (for example, due to the COVID-19 pandemic), or reflect a decrease in cerebrovascular research publications in 2021 more generally. In contrast, the number of publications focusing on obesity in this context show an upward trend, as do epilepsy-related publications.

Most research and applications focused on adults, and middle-aged populations (Figure 1B), while infants were associated with the lowest number of publications. Of publications associated with children (including infants) and those associated with children and young people (which also include adolescents), the most prevalent disease focus is asthma. This is followed by autistic disorder and epilepsy in the child group, while in children and young people, asthma is followed by obesity as the second most prevalent disease focus.

### 4.2. Trends in digital health approaches

**Figure 2:**
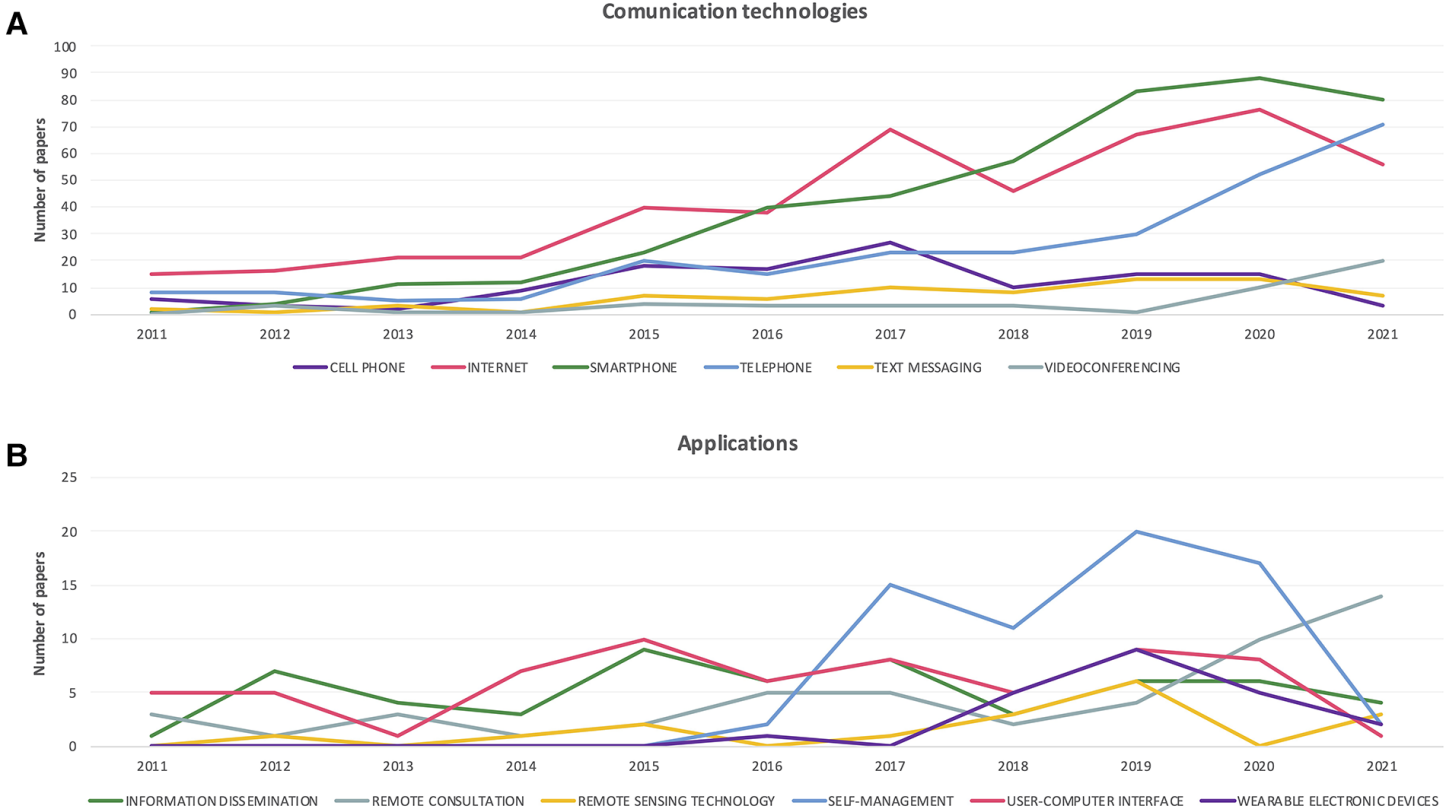
Trends over time in digital health approaches. (**A**) Number of publications by year, associated with different communication technologies and methods. (**B**) Number of publications by year, associated with different digital application types.

In order to capture the type of digital approaches, methods and applications used, PubMed publications were searched for associations with digital-health related MeSH terms (described in Methods); these terms were divided into two groups for presentation purposes (Figure 2).

The term “Telemedicine” was found most frequently in the analysed publications (linked with 1,367 PubMed abstracts), followed by “Internet” (*n* = 466), “Smartphone” (*n* = 444), “Mobile applications” (*n* = 429) and “Software” (*n* = 341). The least common associations were with the terms “Digital divide” (*n* = 8) and “Telerehabilitation” (*n* = 13).

Examination of the trend over time in associations of publications with various digital health related technologies and approaches, reveals an upward trajectory for the communication technologies/methods: “Telephone” and “Videoconferencing”, while other technologies such as “Internet” and “Smartphone” show a slight reduction in the number of associated papers in 2021, compared to previous years (Figure 2A).

In terms of digital health applications, “Remote consultation” shows a steady increase since 2018, while other application-focused approaches such as “User-computer interface” and “Wearable electronic devices” have shown a reduction in published works over the last two years (Figure 2B). Of course, the impact of the COVID-19 pandemic on these trends cannot be gauged within these data.

### 4.3. Digital approaches leveraged for health research and applications

**Figure 3:**
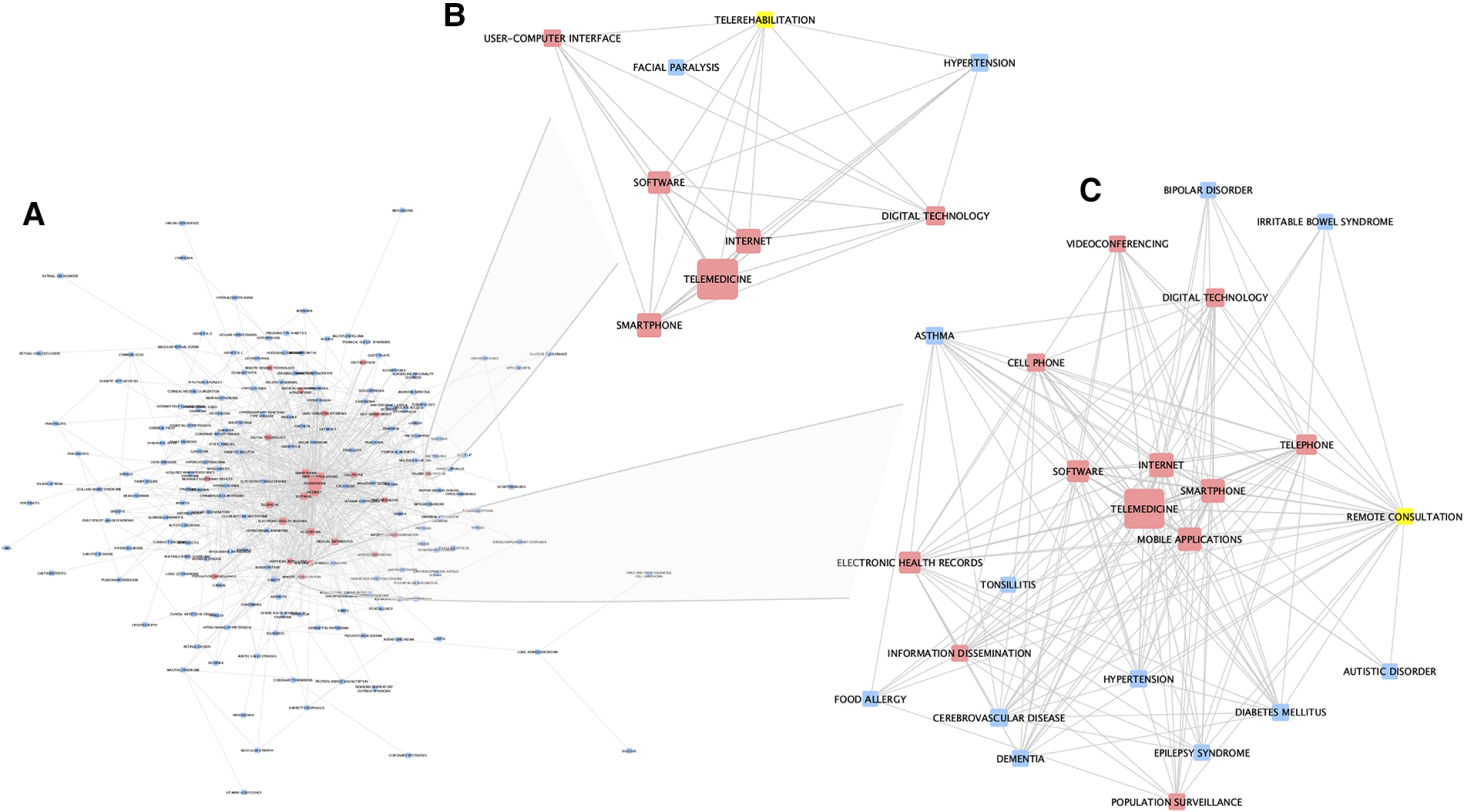
Network analysis of associations between disease and digital-health related approaches, as captured in PubMed publications. (**A**) The whole network (also available in supplementary material). (**B**) Sub-network of first-degree neighbors of the node “telerehabilitation”. (**C**) Sub-network of first-degree neighbors of the node “remote consultation”. Red nodes represent digital health related terms, blue nodes represent diseases, and yellows nodes represent selected nodes used to create the sub-network. The width of edges between nodes corresponds to the number of associations (co-occurrences) between the two entities; the size of the nodes corresponds to the count of that entity within the mined corpus.

Using the co-occurrences of digital health related terms with different diseases, a network of approaches and diseases was created (Figure 3A and Supplementary Material). Cardiovascular disease and telemedicine had the highest number of associations, followed by diabetes and telemedicine, and hypertension and telemedicine. A strong association was also found between obesity, smartphone and mobile applications. Mobile health (mHealth: e.g., smartphones and tablet computers to support and improve health-related services, patient self-management, surveillance, and disease management) shows a strong linkage to online consultations and electronic health care records; this has been suggested to offers major opportunities for improved management in diseases, for example in allergies and asthma (15). Pulmonary telerehabilitation for chronic respiratory diseases, for example, has recently been shown to be beneficial (16).

Further analysis of this network, identified some interesting and differing sub-networks and connections. Figure 3B shows a sub-network created by selecting the node “telerehabilitation” and its first-degree neighbours. This shows links to hypertension and facial paralysis, as well as other digital approaches and technologies. Figure 3C shows a similar sub-network generated by selecting the “remote consultation” node and its first-degree neighbors. Here, links to a wide range of conditions, as well as other digital-health related terms, is demonstrated.

## 5. Discussion

The growth in the use of high-tech digital health devices such as activity trackers, heart rate monitors, and other wearables occurred in parallel with changes in working practices of clinicians (doctors, nurses, allied health professionals, community health workers) to include video-consultations, text message reminders to take medicine or exercise; and other mobile applications and methods for people to access their health information. Empowering telemedicine (remote diagnosis and/or patient treatment), and the availability of molecular phenotyping such as whole-genome sequencing, are driving forces that can augment healthcare. Furthermore, artificial intelligence (AI) approaches, applied to single- or multi-dimensional healthcare data, can enable new approaches in diagnosis, decision support, and treatment.

In this work, we examined the growth and possible links of interest between diseases, age groups, and digital health approaches, methods and applications. By mining textual information captured within biomedical publications associated with at least one UK-based author, in the form of MeSH descriptors, and their co-occurrences, we identified patterns and associations that elucidate the scope of the current landscape of UK-aligned digital health research. Co-authorship across country borders on some submissions to learned journals may occasionally indicate a lower involvement of UK researchers. In general however, rules and guidelines for authorship mean that awareness and commitment to the research is clear for all authors.

### 5.1. Digital health research covers many technologies and disease areas

The most notable growth area in terms of mode of contact remains the ever-increasing use of mobile phones/smartphones. It is likely, the facility and ease of use of phones drives this growth and thus those developing new initiatives should consider the widespread applicability of their technological approach. There are two remote communities where the value of telehealth has been demonstrated. American Indian and Alaskan communities (17) in rural environments have been supported by telehealth since before 2010; clearly the driver for these developments is the remoteness of some communities and this speaks to key catalysts for future developments in the UK. Plainly any such developments must be inclusive and include a range of platforms. The developments possible within the field are exemplified by Médecins Sans Frontières's initiative to address unmet need *via* telemedicine (18). A full and methodical consideration of clinical value from telemedicine across mainly paediatric, surgery, HIV/TB, infectious diseases, internal medicine, nutrition, anaesthesia and obstetrics clearly showed the benefits of the approach (18).

Digital health, it has been argued, has successive developmental phases: development of the approach, implementation *via* first phase studies, validation at greater scale, and development of a more routine patient care orientated approach embedded into health services (19). The Médecins Sans Frontières study demonstrates the value of completing the process. From our review of the literature the scope of disease covered is wide but implementation of validation and roll out to a wider community has been more limited, as assessed by consideration of published works.

We have created an overview of growth areas for digital health research involving the UK, which also indicates those that lag behind. Use of telemedicine in oncology, for example, has not figured highly in our analysis yet outpatient care could potentially be enhanced with new technologies. Because the benefits of telemedicine for oncology patients are clear [tertiary referral and secondary referral centres are often many miles from the patient's home, and cancer disease and treatment decreases mobility (20)] we have taken this forward as a case in point. Benefits and adoption have not been identified in many specific oncology study (21, 22). A systematic review of telehealth research contributions relating to quality of life, pain and depression in cancer patients in 2015 again recognised the benefit of such approaches whilst requiring further larger scale studies for evidence-based advancement (23). A more recent review of literature published between 2000 and 2020, also reached the conclusion that further research is required due to the (unrealised) promise of the approach (24). A similar review concerning haematology and leukaemia concluded there were positive benefits for haematology patients in the use of telemedicine (25). Again, the caveat was clearly stated that more research is required. In patients receiving first-line adjuvant chemotherapy or chemotherapy for the first time, use of a remote symptom management system showed significant reduction in symptom burden (26). In cancer survivors, telehealth is also recognised as a useful approach to providing independence and reassurance following cancer treatment, and engagement with stakeholders as part of future application developments is recommended (27). In summary, digital health was deemed a convenient method that may minimise treatment burden and disruption to those living with cancer.

### 5.2. Consideration of patient requirements

The usual considerations in evidence-based approaches to human health improvement will need to be applied to each aspect of digital health applications, across the range of medical conditions, from mental health through to oncology and inflammatory disease. It has been suggested that telemedicine will help clear the backlog of those concerned about cancer who contact primary care. In the UK a face-to-face appointment is only offered, for guidance proposes, after a video, telephone, or electronic consultation (28). Plainly, quality and safety of care need to be maintained, whilst also ensuring remote consultations address the patient's needs. This can only be assured by further research as proposed above. In a relatively rare cancer, sarcoma, such research has reported positive feedback about the telemedicine approach from clinicians and their patients (29). There was less positivity about virtual consultations for lung cancer patients and their clinical teams, albeit this was coloured by the COVID-19 pandemic (30).

### 5.3. Clinician requirements

The Ipsos Digital Doctor survey (31) indicated that in a COVID-19 era only 8% of UK clinicians saw no advantage to practicing medicine virtually. The clinicians' perspective was also that it offers greater flexibility is offered to patients (69% agreed). The explosion in on-line medicine and telephone consultations was also shown in this survey. Primary care consultations *via* telemedicine rose from 1% to 51% before and after COVID 19 pandemic respectively. In oncology, the figures rose from 1% to 73% and in immunology from 1% to 65%. Thus, the era of telemedicine is with us and there is an active adoption of the approach in oncology which is yet to be appropriately assessed. Nonetheless, a consumer-based assessment of the Babylon GP tool, which gives users access to a doctor, therapist or specialist through video or phone consultation, found the tool convenient and effective (32). Importantly, however, those who were isolated or housebound were less satisfied with the service, exemplifying the need for a human touch in aspects of medical services. Babylon are partnering with the Royal Wolverhampton National Health Service (NHS) Trust to deliver an integrated health app with no investment from the NHS. This is a sign of the disruptive effect technologies in this area may have in the future.

However, NHS primary care in the UK is the foundation of the healthcare system. Recently primary care in England was charged with delivering seven service specifications. Structured medication reviews, enhanced health in care homes, and supporting early cancer diagnosis began in 2020/21 (https://www.kingsfund.org.uk/publications/primary-care-networks-explained). Anticipatory care (with community services), personalised care, cardiovascular disease case-finding, and, tackling inequalities, start soon. The use of telemedicine in meeting some of these requirements is to be seen as an advantage. Negative impact on the doctor–patient relationship, the quality of the physical examination, and the quality of care have all been highlighted as issues in telemedicine as well as the need for appropriate infrastructure and investment to work effectively (33). Consideration of Figure 2 demonstrates the infrastructural requirements in this arena. This inevitably leads to questions of participation. In 2020, 96% of households in Great Britain had internet access but the age group with greater disease burden, - households containing one person >65 years—reported lower internet connection (80%). Figure 1B clearly shows the patient or volunteer age in respect of digital health publications is highly proportioned in the elderly/aged section of society yet these are the individuals with lower internet access and proven lower skill levels in handling new technology. While telemedicine services can be accessed *via* a range of different devices, only 65% of the over 65s in the UK have a smartphone (https://www.statista.com/statistics/300402/smartphone-usage-in-the-uk-by-age/) and over 40% of those aged 75 + do not use the internet (34).

### 5.4. Hard to reach populations for telemedicine need consideration

Mitigating against exclusion of hard-to-reach populations and the elderly will be important. The argument for telemedicine preceding any face-to-face appointment needs to bear these factors in mind. Consideration of the data presented in Figures 1, 3 shows cerebrovascular disease, obesity, diabetes and other diseases of older people as being key areas for UK-based telemedicine research. Ergo, accommodating the >65s in digital health strategies is necessary and appropriate at least until technological skills penetrate effectively into all age groups. It has been suggested that increased usage of telemedicine can be achieved *via* training, public lectures and adapting apps for use by the elderly (35, 36). This then goes back to the need for the four stages of research and product development in telemedicine to be more rapidly developed.

### 5.5. Adoption of digital health practices: customers, concerns and COVID-19

In the UK, the NHS is the key customer for digital health products and IT (with a budget of £5 billion) compared to a private sector investment of about £250 million. A 2015 report commissioned by the Office of Life Sciences on digital health in the UK has identified the UK as an early adopter of several areas in the digital health market, such as telecare and primary care electronic health record systems (37). In part, this fulminating activity is due to intensive academic interest in major universities such as Imperial College London, Oxford, Cambridge and Edinburgh. This has been driven by top-down policy initiatives and government grant funding. Further, the report identifies areas where opportunities exist for growth, such as in the adoption of secondary care electronic health records, mHealth and data analytics. Analysis by Bertelsmann Siftung (38) reported the UK as sixth globally in terms of digital health policy development, use of data and digital readiness. This report highlights the key needs for development in this area: *digital transformation needs political leadership and coordination. Successful countries are characterised by a trio of effective strategy, political leadership and coordinating national institutions, i.e., “agencies for digital health”. The process of digitalization in successful countries is health benefit-oriented and is implemented in pragmatic steps*. The monolithic nature of the NHS has been a blessing and an issue in terms of adoption of digital health. Many key performance indicators of successful adoption are met in the NHS. However, the significant independence of the primary care networks allows these healthcare delivery frontline institutions to be selective in which digital health applications to use for their services. In a very busy healthcare economy this can be a drag on innovation. An NHS review of innovation (39) has described the growth in uptake of telemedicine, phone apps, automated image interpretation, use of wearables and advanced use of genomics. Published before the COVID-19 pandemic, what is noteworthy is telemedicine somewhat lagging behind adoption of other approaches (50% of NHS workforce affected by 2030). The pandemic clearly changed that rate. In Figure 2 the major growth area recently observed is remote consultation, a necessary by-product of lockdown procedures and self-isolation. The corollary of this is more than 22 million people now use the NHS App. Likely, the rapid increase in rate of usage is down to the COVID Pass system and the means to access it through the app. There were 18 million registrations in 2021, and more than 140 million COVID Passes generated through the NHS App and NHS website since the service was added in May of that year. The NHS COVID Pass, until recently, has been used for obtaining COVID-19 vaccination and recovery status, enabling travel or event attendance. This catalyst for change then leads to altered habits in respect of prescription orders and appointment bookings. For example, NHS 111 online enables UK citizens to get urgent healthcare online. Before the COVID-19 outbreak, NHS 111 online recorded about 10,000 users/day. In early 2020, NHS 111 online had on average 548,000 users/day. Whilst the number of online enquiries have and will fall, the means of accessing health care digitally have been altered in practice, and in the perception of the public (40).

In other words, the activation energy required for changing behaviours with respect to apps has likely been modulated by the pandemic. This then enables further initiatives on digital health to potentially proceed more rapidly. This does not however, diminish the legal, ethical, safety, access and privacy issues that need to be addressed post COVID-19, neatly summarised by Budd et al. (41). Other examples of healthcare improvements that can be gained include an app-based intervention tested in a randomised control study in patients with obesity, diabetes and hypertension that led to improved outcomes (42). Additionally systematic reviews of self-help apps have been performed (43, 44). Thus, the benefits of systematically proceeding through the four stages of telemedicine and digital health development are clear in specific examples and now need further impetus in specific disease areas.

Understanding the rate of pace of change in the size and the shape of the digital health economy, and progress in further adoption can be, in part, achieved by measuring research outputs and *via* Cochrane reviews. This is because peer reviewed publications remain a form of currency in acceptance of changes in healthcare practice. Abernethy et al. (45) have defined the framework for successful adoption which highlights the interconnected processes required, inclusive of full exposure to data and findings. Nonetheless a consideration of patents and new companies also enables further information gathering in this arena. This is as opposed to measures from companies (e.g., their numbers, sizes, capitalisation, products) as many start-ups exist in this area who are not impacting on healthcare practice and have not proceeded through the all-important latter stages of development. This has led to some funding initiatives such as those sponsored by the National Institute for Health Research in the UK (e.g., Artificial Intelligence in Health and Care Award). Publications show (in general) peer reviewed research and trial data as opposed to advertisement and investor briefings. Thus, we have generated an objective understanding of the Digital Health landscape aligned to the UK and can identify where it will impact in healthcare in the future. Nonetheless, summaries of growth in the sector are available and of value to understanding the underlying technology and market (see https://www.beauhurst.com/blog/top-healthcare-companies-and-investment-trends/).

### 5.6. Limitations and future work

It should be noted that some of the trends observed by this study, especially in association with digital health technologies, could be, at least in part, a reflection of the evolution of the language researchers use to describe technology or advances in the technology itself. For example, a switch from using the terms “cell-phone” or “mobile-phone”, to “smartphone”. This in turn could skew some of the conclusions drawn. Further, UK activity was determined by inclusion of at least one UK-based author, even in cases where research may have been led in other countries. There is the caveat that some articles considered are inclusive of international collaboration and globalisation phenomena which may slightly alter data interpretation. In future work we will assess specific areas identified as very active in the UK and consider how healthcare can be ameliorated by improved education and research within the dynamic growth buds of Digital Health in the UK.

One shortcoming is that industry do not necessarily publish their tools or findings to protect intellectual property. Nonetheless our aim has been fulfilled with the analysis of works published in learned journals.

## 6. Conclusion

Through the mining of associations in published works, we have gained an understanding of the progressive development of- and trends in digital health using quantitative measures on peer reviewed publications. Importantly, the method includes an analysis of NHS activity which is fundamentally important in the UK to adoption and usage of new technologies at scale. This methodology has thrown up key points as described in the discussion. The data here and produced elsewhere, using this methodology, allow critical analysis of a field of research. Thus our tool can be used iteratively to monitor trends in the field. Furthermore, as neologisms are an aspect of this field, they can easily be included in the searches to maintain an up-to-date analysis of trends for patients, practitioners, and industry.

## Data Availability

Publicly available datasets were analyzed in this study. The data used for this study was accessed through the PubMed database, on December 27th, 2021 (see methods) and is publicly available online. All analysis results are available in the Supplementary Material.
