# Supplementary material for: Identifying developments over a decade in the digital health and telemedicine landscape in the UK using quantitative text mining
Source: Front Digit Health. 2023 Apr 17;5:1092008. doi: 10.3389/fdgth.2023.1092008 (PMC10149860; doi:10.3389/fdgth.2023.1092008)
Supplement: Supplementary file 1 [file Datasheet1.zip › supplementary/PubMed_Search.rtf]

(((((((("mobile applications"[All Fields]) OR ("digital health"[All Fields])) OR ("telemedicine"[All Fields])) OR ("digital intervention"[All Fields])) OR ("health app"[All Fields])) OR ("medical informatics"[All Fields])) OR ("health informatics"[All Fields])) OR ("digital technology"[All Fields])) AND (("united kingdom"[Affiliation]) OR ("UK"[Affiliation]))# 9199 hits, as of December 27 2021, since Jan’ 1st 2011, with abstract available
