# Supplementary figures and images for: Identifying developments over a decade in the digital health and telemedicine landscape in the UK using quantitative text mining
Source: Front Digit Health. 2023 Apr 17;5:1092008. doi: 10.3389/fdgth.2023.1092008 (PMC10149860; doi:10.3389/fdgth.2023.1092008)

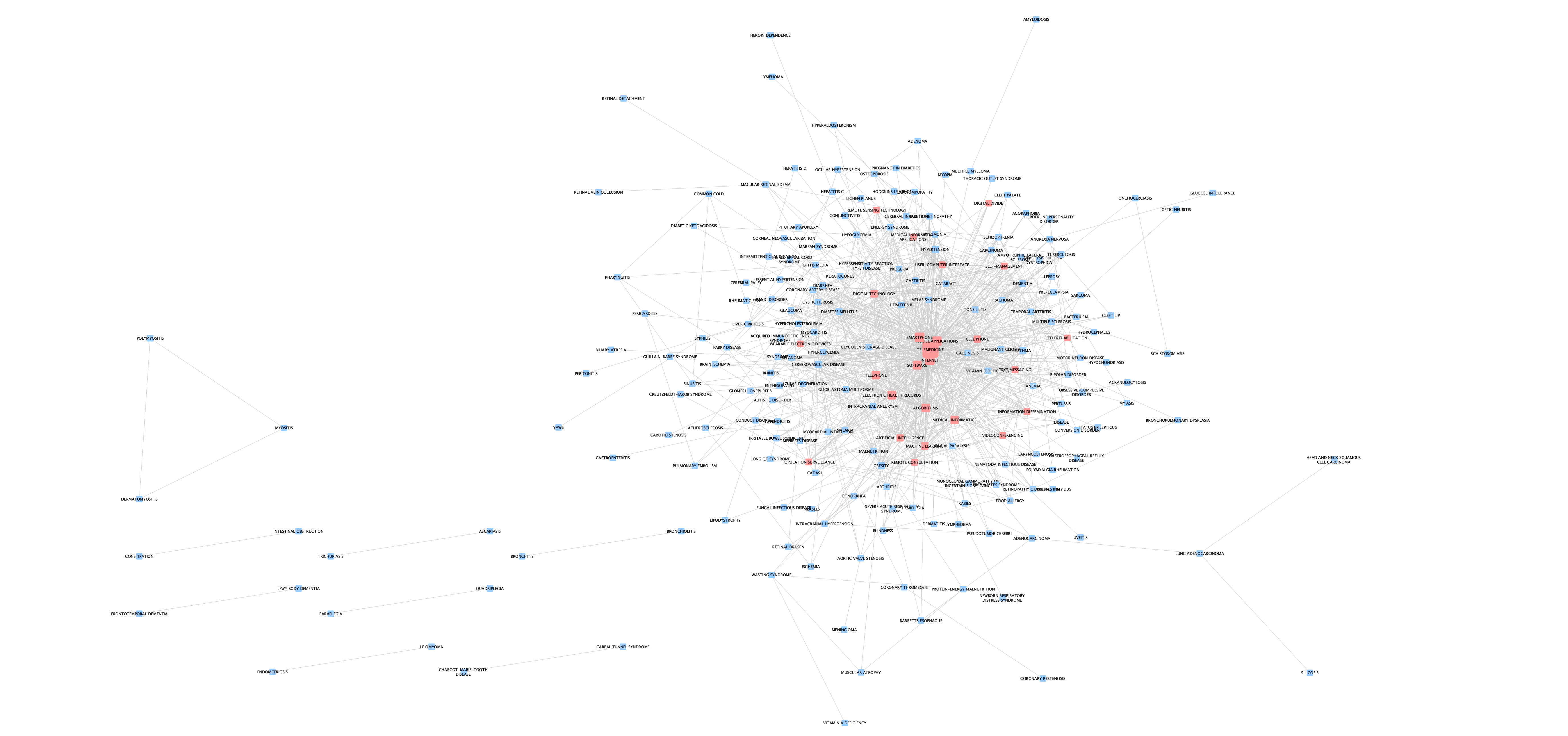

Supplement: Supplementary file 1 [file Datasheet1.zip › supplementary/digital_disease_network.png]
